# Enhanced In Vitro Biocompatible Polycaprolactone/Nano-Hydroxyapatite Scaffolds with Near-Field Direct-Writing Melt Electrospinning Technology

**DOI:** 10.3390/jfb13040161

**Published:** 2022-09-23

**Authors:** Zhijun Chen, Yanbo Liu, Juan Huang, Han Wang, Ming Hao, Xiaodong Hu, Xiaoming Qian, Jintu Fan, Hongjun Yang, Bo Yang

**Affiliations:** 1 State Key Laboratory of Separation Membranes and Membrane Processes, School of Textile Science and Engineering, Tiangong University, Tianjin 300387, China; 2 State Key Laboratory of New Textile Materials and Advanced Processing Technologies, School of Textile Science and Engineering, Wuhan Textile University, Wuhan 430200, China; 3 School of Materials Science and Engineering, Wuhan Textile University, Wuhan 430200, China

**Keywords:** melt electrospinning, near-field direct-writing, composite scaffolds, biocompatibility

## Abstract

Polycaprolactone (PCL) scaffold is a common biological material for tissue engineering, owing to its good biocompatibility, biodegradability and plasticity. However, it is not suitable for osteoblast adhesion and regeneration of bone tissue due to its non-biological activity, poor mechanical strength, slow degradation speed, smooth surface and strong hydrophobicity. To improve the mechanical properties and biocompatibility of PCL scaffold, the PCL/nHA scaffolds were prepared by melting and blending different proportions of nano-hydroxyapatite (nHA) with PCL by the near-field direct-writing melt electrospinning technology in this study. The morphology, porosity, mechanical properties and in vitro biocompatibility of the PCL/nHA scaffolds were studied. The results showed that when the proportion of nHA was less than or equal to 25%, PCL/nHA composite scaffolds were easily formed in which bone marrow mesenchymal stem cells proliferated successfully. When the proportion of nHA was 15%, the PCL/nHA composite scaffolds had excellent structural regularity, good fiber uniformity, outstanding mechanical stability and superior biocompatibility. The PCL/nHA composite scaffolds were ideal scaffold materials, which would broaden their applications for bone tissue engineering.

## 1. Introduction

Polycaprolactone (PCL) is a kind of synthetic polyester biological material, characterized by high crystallinity and good flexibility. It is an FDA-approved material for tissue engineering biomaterials due to its good biocompatibility, biodegradability and plasticity, but it is not suitable for osteoblast adhesion and regeneration of bone tissue by itself owing to its non-biological activity, poor mechanical strength, slow degradation speed, smooth surface and strong hydrophobicity [1,2]. In general, it is used in combination with Hydroxyapatite (HA) to enhance osteogenic properties or biomechanical strength [3,4]. HA belongs to the ceramic inorganic biomaterial group, and its chemical composition and crystal structure are similar to natural bone minerals [5,6]. When HA is used alone, it has some disadvantages such as low fatigue strength, poor flexibility and plasticity, easy agglomeration and a sudden release effect [7,8]. Therefore, it is often combined with PCL to obtain more excellent biological properties [9,10,11]. 

In many biomaterials studies for bone repair, there are many processing methods of the PCL/nHA system commonly used such as electrospinning, three-dimensional (3D) printing and dissolution etching, etc. [12,13,14,15,16,17,18]. However, these processing methods have some shortcomings. Electrospinning brings organic solvents into the system, and the fiber membrane is a two-dimensional system, which is not suitable for cell growth [19,20,21,22,23]. Three-dimensional printing is a common method of additive manufacturing, resulting in coarse products with low accuracy, which require post-processing [24,25,26,27]. The process of dissolution etching is long, low efficiency and it is difficult to control the structure of material holes [28,29,30]. The PCL/nHA system urgently needs to find a high processing efficiency, no pollution, and simple process, with a controllable structure of the molding process [31,32,33].

The near-field direct-writing melt electrospinning (NFDWME) technology avoids the instability of solvent volatiles and the pollution of toxic solvents and prepares ordered micro-nano fibers, which has become a hot spot in the field of electrospinning research and has a certain prospect in the clinical application of tissue engineering scaffolds [34]. Its main principle is to introduce a high-voltage electrostatic field to 3D-printing equipment. The receiving platform can be precisely controlled. That is, based on the straight line of the melt spinning jet, when the distance between the receiving plate and the spinning needles is shortened, the orderly deposition of fibers can be achieved by the stable movement of molten fibers along with the platform, and an orderly arrangement of micro-nano fiber structure can be obtained accordingly [35,36,37].

On the basis of previous studies, PCL and nHA were used to prepare PCL/nHA scaffolds with different proportions by NFDWME technology. The high PCL ratio is beneficial to the forming and structure control of composite scaffolds. However, the mechanical properties and osteogenic ability of composite scaffolds may not meet the standard of bone substitutes. The addition of nHA can improve the mechanical strength and bone conduction properties of a composite scaffold, but the high proportion of nHA will lead to difficulty in forming the composite scaffold. Therefore, the appropriate ratio of PCL to nHA is the key to constructing good bone conductivity of PCL/nHA scaffolds. This study investigated the physical properties and in vitro biocompatibility of PCL/nHA scaffolds with different proportions, laying a foundation for the preparation of biological scaffolds for bone repair by NFDWME technology in clinical application.

## 2. Experimental Section

### 2.1. Experimental Materials

PCL with relative molecular weight of about 80,000 Da was prepared by Shanghai Aladdin Biochemical Technology Co., Ltd., Shanghai, China. nHA with relative molecular weight of about 500 Da, purity not less than 97% and particle size less than 100 nm was manufactured by Shanghai Aladdin Biochemical Technology Co., Ltd. Ethanol, analytical reagent, was purchased from Sinopharm Chemical Reagent Co., Ltd., Shanghai, China. Phosphate buffer solution (PBS), biological grade, was provided by HyClone. Trypsin, biological grade, was prepared by HyClone. Cell Counting Kit-8 (CCK-8), biological grade, was manufactured by Biosharp. 4′,6-diamidino-2-phenylindole (DAPI) fluorescent dye, biological grade, was purchased from Shanghai Biyuntian Biotechnology Co., LTD., Shanghai, China. The 4% paraformaldehyde, biological grade, was provided by Biosharp. Calcein-AM/PI live and dead cell detection kit, biological grade, was prepared by Shanghai Biyuntian Biotechnology Co., LTD. The mouse bone marrow mesenchymal stem cells (mBMSCs) were provided by the Stem Cell Bank of the Chinese Academy of Science, Beijing, China.

### 2.2. Preparation of PCL/nHA Scaffolds with Different Proportions

**Figure 1:**
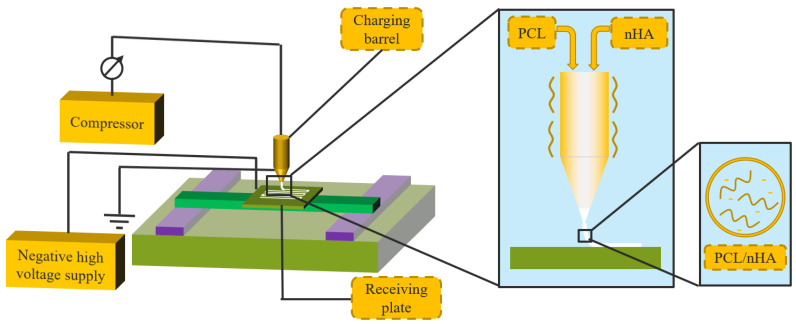
Schematic diagram of preparation of PCL/nHA scaffolds with different proportions.

PCL/nHA scaffolds with different proportions were prepared by the NFDWME equipment, as shown in Figure 1. The raw materials were added from the barrel, heated and fused. The process parameters were set to ensure that PCL/nHA scaffolds were prepared by fiber deposition in the form of square wave printing. The mass ratios of PCL to nHA were 100:0, 95:5, 90:10, 85:15, 80:20 and 75:25. Six comparative samples were prepared and designated as Pure PCL scaffold, PCL–5%nHA scaffold, PCL–10%nHA scaffold, PCL–15%nHA scaffold, PCL–20%nHA scaffold, PCL–25%nHA scaffold and PCL–30%nHA scaffold, respectively. The specific experimental parameters were as follows: the spinning voltage was −4 kV, the receiving distance was 4 mm, the moving speed of receiving plate was 5 mm/s, the melt temperature was 130 °C, the fiber spacing was 1 mm, the number of layers was 3, the number of square waves was 10 times, the inner diameter of the needle was 0.5 mm, and the pressure of air pump connected to the barrel was 0.2 MPa.

### 2.3. Testing

#### 2.3.1. Macroscopic Morphology

The sample size of PCL/nHA scaffolds with different mass ratios was 2 cm × 2 cm, and the macroscopic morphology of all samples was photographed with a camera.

#### 2.3.2. Microscopic Morphology

PCL/nHA scaffolds with different mass ratios were cut with a scalpel. After gold spraying, the surface morphology and Energy Dispersive Spectroscopy (EDS) of the samples were observed by scanning electron microscopy (SEM, JSM-6510LV, Tokyo, Japan). A total of 50 fibers for each sample were selected randomly by Image J, and the diameter was measured and averaged afterward.

#### 2.3.3. Sectional Morphology

The sections of PCL/nHA scaffolds with different mass ratios were cut vertically down from the section with a scalpel. The sectional morphology of the samples was observed by digital microscopy. 

#### 2.3.4. Porosity

The length, width, and height of each sample cut with a scalpel were measured, and the corresponding volume was recorded as *V*_1_. A certain amount of ethanol was added to a graduated cylinder, and the volume of ethanol was recorded as *V*_2_. The sample was immersed in ethanol for 10 min to ensure no bubbles on the sample surface. At this moment, the volume was recorded as *V*_3_. The porosity (*ε*) can be calculated by Equation (1).

*ε* = [1 − (*V*_3_ − *V*_2_)/*V*_1_] × 100% (1)

#### 2.3.5. Mechanical Performances

The mechanical performances of PCL/nHA scaffolds with a length of 20 mm and width of 5 mm were investigated by an electronic universal testing machine (MTS Systems CO., LTD E44.104, Shanghai, China) with a gauge length of 10 mm and tensile speed of 10 mm/min. Each reported value was the average of five valid specimens.

#### 2.3.6. Fiber Peeling Strength

The molten polymer extruded from spinning needle formed fibers and bonded into the previous layer. The adhesion degree between fibers in layers determines the sample strength and is generally characterized by the peeling force between a single fiber and the sample. A single fiber was picked out with tweezers, and held at two ends by the upper clamp and the lower clamp of electronic universal testing machine (MTS Systems CO., LTD E44.104, Shanghai, China), respectively. The fiber was peeled from the sample surface with the drawing speed of 10 mm/min, and the maximum tensile force was obtained during the process of the fiber completely detaching from the sample.

#### 2.3.7. In Vitro Cytocompatibility of Composite Scaffolds

(1) Cell seeding

The mBMSCs were resuscitated and passed to the sixth generation for the experiment. The sixth generation mBMSCs were digested with trypsin and diluted into a suspension with a cell concentration of 1 × 10^5^ mL^−^^1^. The previously sterilized PCL/nHA scaffolds were placed in a 96-well culture plate, and the mBMSCs were seeded on the composite scaffolds (1 × 10^4^ cells/well). The culture plate was placed in a cell culture box with 5% CO_2_ at 37 °C and the suspension was changed every 3 days.

(2) Cell proliferation activity detection

The culture plate with 96 wells for each sample was taken out on the 1st, 3rd and 5th day after inoculation. A total of 100 μL of CCK-8 solution was added to each well of the culture plate, which was then placed in an incubator for 2 h. Subsequently, the culture plate was taken out and the absorbance value of each well at 450 nm was detected by a multi-function multimode reader.

(3) Live/Dead staining

Live/Dead staining was performed on the 1st, 3rd and 5th day of composite scaffold culture. A total of 2 µL of calcein-AM and 1 µL of propidium iodide were added to 2 mL of PBS to obtain the staining solution. First, the samples were sucked out from the wells. Second, PBS was employed to rinse the samples 3 times. Third, appropriate staining solution was added to the wells, which were incubated at 37 °C without light for 30 min. The fluorescent microscope was used to take photos and observe the state of the cells.

(4) Cell morphology observation

The cell morphology was observed on the 3rd day of composite scaffold culture. The cells were orderly fixed with 4% paraformaldehyde for 15 min, cleaned with PBS twice, treated with 0.3% TritonX-100 solution for 10 min, incubated with FITC-photoleptide working solution at 37 ℃ for 30 min, cleaned with PBS twice, nucleated with DAPI solution for 5 min, cleaned with PBS twice and inverted on the glass slide dripping with sealing tablets. The cell morphology was observed by a fluorescence microscope.

(5) Main outcome measures

The main outcome measures were proliferation and growth of mBMSCs on the composite scaffolds.

(6) Statistical analysis

GraphPad Prism software was used for statistical analysis and all data were expressed as x ± s. Statistical analysis of variance was performed on the measured data. Single-factor ANOVA was used for comparison between multiple groups, and P < 0.05 was considered a significant difference.

## 3. Results and Discussion

### 3.1. Macroscopic Morphology of PCL/nHA Scaffolds with Different Proportions

**Figure 2:**
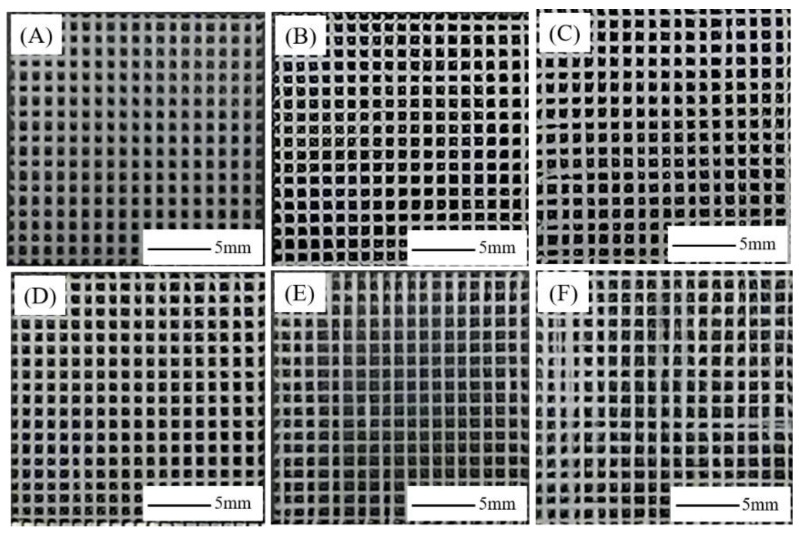
Macromorphology of PCL/nHA scaffolds with different proportions: (**A**) Pure PCL, (**B**) PCL–5%nHA, (**C**) PCL–10%nHA, (**D**) PCL–15%nHA, (**E**) PCL–20%nHA, (**F**) PCL–25%nHA.

The macroscopic morphology of PCL/nHA scaffolds with different proportions is shown in Figure 2. The transverse and longitudinal fibers in samples Figure 2A–C were arranged in an orderly manner with uniform fiber thickness. The fibers in sample Figure 2D were coarse, the intermediate fibers in sample Figure 2E were fine, and the edge fibers were thick. From Figure 2F, the fiber regularity was poor, the upper and lower fibers were misaligned, and the uniformity of fiber fineness was unsatisfactory. The high nHA ratio of the PCL–30%nHA scaffold affected the fluidity of the melt, causing the needle to be blocked, therefore the molding process was not as smooth. The reason behind this was that due to the large nHA ratio, the melt fluidity was poor and the melting amount per unit time was small. When the needle moved to the middle, the fiber was subject to a certain amount of drag force, resulting in serious fiber refinement. When the needle moved to the edge, there was a deceleration process, leading to obvious fiber thickening.

### 3.2. Microscopic Morphology of PCL/nHA Scaffolds with Different Proportions

**Figure 3:**
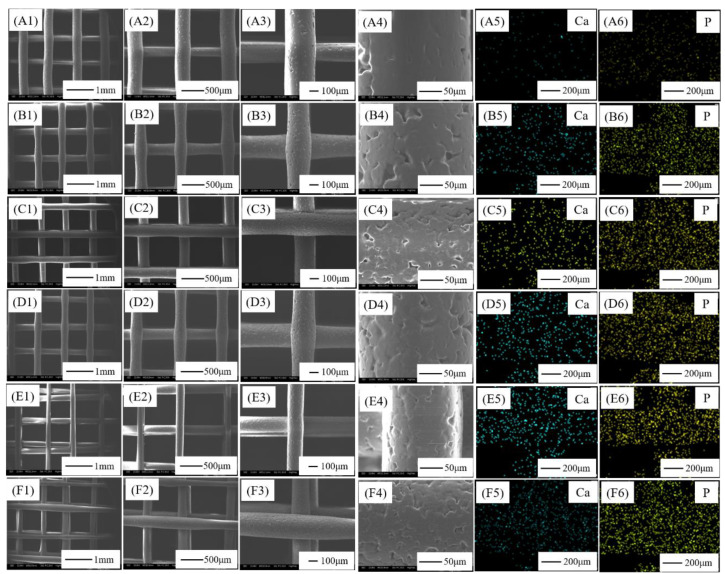
Microscopic morphology and EDS of PCL/nHA scaffolds with different proportions: (**A**) Pure PCL, (**B**) PCL–5% nHA, (**C**) PCL–10% nHA, (**D**) PCL–15% nHA, (**E**) PCL–20% nHA, (**F**) PCL–25% nHA; (**A1**–**F1**) magnified 30 times, (**A2**–**F2**) magnified 50 times, (**A3**–**F3**) magnified 100 times, (**A4**–**F4**) magnified 500 times, (**A5**–**F5**) Ca energy spectrum, (**A6**–**F6**) P energy spectrum.

The microscopic morphology, Calcium (Ca) energy spectrum and Phosphorus (P) energy spectrum of the PCL/nHA scaffolds with different proportions was observed by SEM, as shown in Figure 3. The SEM results showed that all fibers were arranged horizontally and vertically, which was formed by square wave printing. The composite scaffolds had a regular structure and clear pores with an average pore size of 500–800 μm formed by the fibers arranged in horizontal and vertical directions. The fibers in the upper and lower layers of samples Figure 3A–D are orderly, while the fibers in the upper and lower layers of samples Figure 3E,F are not completely orderly. It can be seen from the 100 times magnification of Figure 3(A3)–(F3) that the ratio of PCL and nHA is different and the fiber diameter is different, which will be further analyzed quantitatively later. Figure 3(A4)–(F4) is the micrograph with a magnification of 500 times. The fiber surface is clear and the grooves formed by cooling crystallization can be seen. nHA particles can be seen on the surface of Figure 3(D4)–(F4) fiber, which was conducive to cell adhesion and growth. The ratio of PCL to nHA determined the fluidity of the melt, which affected the regularity, diameter and microstructure of the fiber.

Figure 3(A5)–(F5),(A6)–(F6) are the Ca energy spectra and P energy spectra of the PCL/nHA scaffolds with different proportions, respectively. It can be seen from the pictures that there were Ca elements and P elements on the scaffolds, and there were a few pixels of Ca and P elements on the surface of the Pure PCL scaffolds but almost none, probably because there was a very small amount of nHA residue in the charging barrel during repeated processing, which was brought into the Pure PCL sample. In Table 1, quantitative analysis of carbon (C), oxygen (O), Ca and P elements in the PCL–15%nHA and PCL–20%nHA scaffolders showed that the content of nHA in the PCL–20%nHA samples was higher than that in the PCL–15%nHA samples, and the content of Ca and P elements in the former was also higher than that in the latter. Corresponding to the number of element pixels in the picture, with the increase in nHA content, the pixel points of the Ca element and P element in the sample increased.

### 3.3. Sectional Morphology of PCL/nHA Scaffolds with Different Proportions

**Figure 4:**
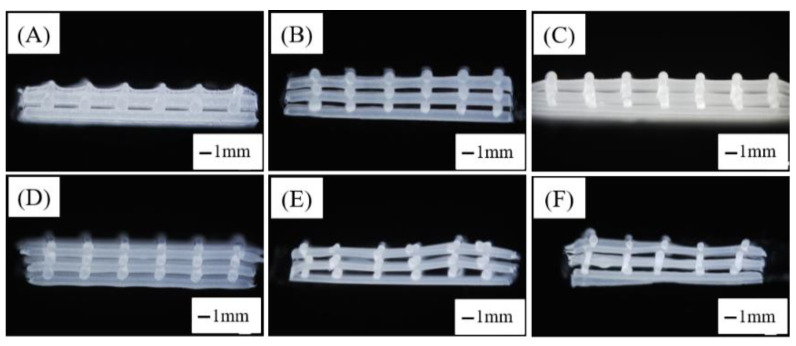
Sectional morphology of PCL/nHA scaffolds with different proportions: (**A**) Pure PCL, (**B**) PCL–5%nHA, (**C**) PCL–10%nHA, (**D**) PCL–15%nHA, (**E**) PCL–20%nHA, (**F**) PCL–25%nHA.

The sectional morphology of the PCL/nHA scaffolds with different proportions is shown in Figure 4. As can be seen from the figure, the pore structure formed between the horizontal and vertical fibers of samples Figure 4A–D was regular, and there were certain differences in thickness, pore size and fiber diameter. Meanwhile, the fibers of samples in Figure 4E,F were not neatly deposited, resulting in an uneven thickness of samples. The upper and lower layers of sample Figure 4F were obviously dislocated, causing uneven fiber thickness and irregular pore structure. The ratio of PCL to nHA was different, which affected the diameter of the fiber and the regularity of the upper and lower fibers during molding, and thus the rules of the holes formed by the section.

### 3.4. Physical Properties of PCL/nHA Scaffolds with Different Proportions

In order to understand the physical properties of the PCL/nHA scaffolds with different proportions, the average fiber diameter, diameter CV, porosity, tensile strength and fiber peeling force were investigated, the experimental results are shown in Table 2. With the increase in the nHA ratio, the melt fluidity became worse and the fiber diameter of the composite scaffolds decreased. The average diameter of the Pure PCL fiber was 398 μm, and that of the PCL–25%nHA fiber was only 212 μm, which was about half that of the former. The ratio of PCL to nHA had a great influence on the fiber diameter of the composite scaffolds. The diameter CV value increased with the proportion of nHA in the PCL/nHA scaffolds. The diameter CV values of the PCL–20%nHA and PCL–25%nHA scaffolds were 15.81% and 23.77%, respectively, larger than those of other samples, manifesting that the fiber uniformity of both samples decreased markedly. This was consistent with the previous results of macroscopic morphology analysis, microscopic morphology analysis and sectional morphology analysis. The porosity of composite scaffolds ranged from 70% to 82%, the proportion of nHA had little effect on the porosity of the composite scaffolds, and the porosity increased slightly with the increase in the proportion of nHA. The tensile strength and fiber peeling force of the composite scaffolds decreased with the increase in the proportion of nHA, and the trend of decline was obvious. When the proportion of nHA reached 20% and 25%, the tensile strength and fiber peeling force decreased sharply. The ratio of PCL and nHA determined the fluidity of melt, affected the molding process of the PCL/nHA scaffolds, and had a significant impact on the physical properties of the PCL/nHA scaffolds. It determined the thickness and uniformity of fibers and further affected the mechanical properties of the scaffolds and the binding force (peeling force) between fibers.

### 3.5. Proliferation of mBMSCs on Composite Scaffolds

**Figure 5:**
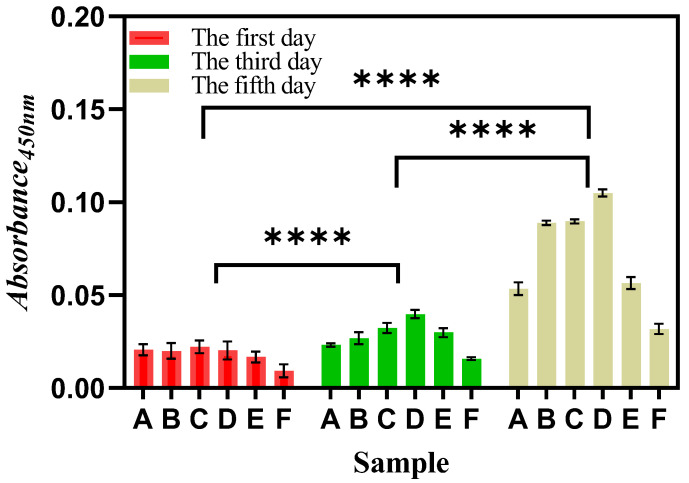
Proliferation of mBMSCs on PCL/nHA scaffolds with different proportions: (A) Pure PCL, (B) PCL–5%nHA, (C) PCL–10%nHA, (D) PCL–15%nHA, (E) PCL–20%nHA, (F) PCL–25%nHA. “****” represented the degree of significance was extraordinary.

The CCK-8 method was used to detect cell proliferation on the PCL/nHA scaffolds. The mBMSCs were seeded in six groups of scaffold materials and the absorbance of the cell growth solution at 450 nm spectrum was measured after 1, 3 and 5 days of cell culture, respectively. As shown in Figure 5, with the extension of culture time, compared with the first, third and fifth days, the absorbance increased, indicating that mBMSCs could proliferate effectively on the six groups of composite scaffolds. By comparing the absorbance of the PCL/nHA scaffolds on day 1, day 3 and day 5, the cell proliferation was different with different PCL/nHA proportions, and the degree of significance was extraordinary. For example, the absorbance of the PCL–15%nHA sample was higher than that of other groups, which preliminarily indicated that the growth of cells on this scaffold was better than that of other groups. The absorbance of the PCL–20%nHA and PCL–25%nHA samples was lower than that of the Pure PCL samples, which may be due to the poor regularity and uneven fiber thickness of the samples, which affected the normal growth of cells. From the above results, it can be concluded that the PCL–15%nHA sample was more suitable for the growth of mBMSCs. 

### 3.6. Live/Dead Staining of mBMSCs on Composite Scaffolds

**Figure 6:**
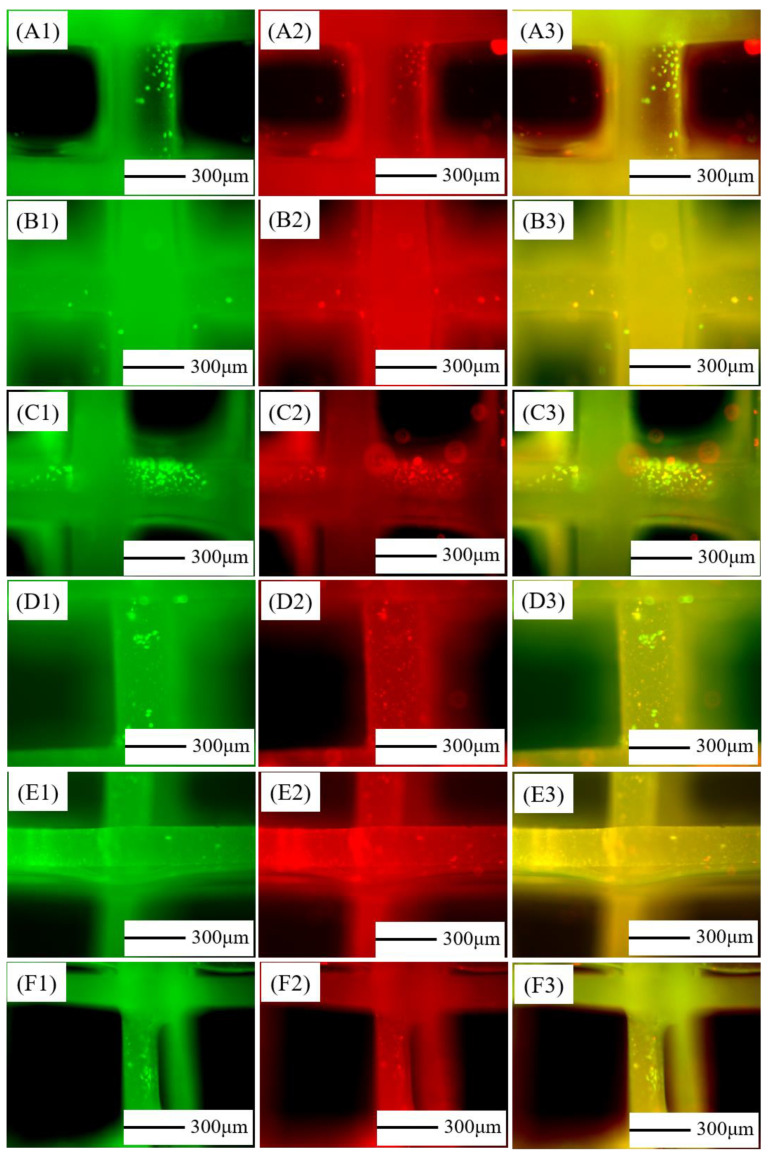
Growth of mBMSCs on the surface of PCL/nHA scaffold with different proportions for three days: (**A**) Pure PCL, (**B**) PCL–5% nHA, (**C**) PCL–10% nHA, (**D**) PCL–15% nHA, (**E**) PCL–20% nHA, (**F**) PCL–25% nHA; (**A1**–**F1**) live cells staining, (**A2**–**F2**) dead cells staining, (**A3**–**F3**) live/dead cells staining.

The samples of cells cultured on the PCL/nHA scaffolds for three days were stained for live cells as shown in Figure 6(A1)–(F1), dead cells as shown in Figure 6(A2)–(F2), and the combination of live/dead cells as shown in Figure 6(A3)–(F3). From the pictures, there were both live and dead cells on the scaffolds. The cell numbers from the PCL–10%nHA sample were relatively large and the ratio of live cells to dead cells was about 1:1. The cells on the PCL–15%nHA scaffold were scattered, the number of live cells was more than dead cells, and the cell viability was good. The fibers of the PCL–20%nHA and PCL–25%nHA scaffolds were fine, uneven in thickness, and a few cells were scattered sporadically. It can be speculated that the regularity of the scaffold and the morphology of the fibers had an effect on the growth of mBMSCs. When the proportion of nHA was moderate and the scaffold was regular, the sample was suitable for the growth of mBMSCs. The PCL–15%nHA scaffold was superior in the six groups of experimental samples. Compared with the 3.2 EDS analysis, there were more Ca element pixels on the surface of the PCL–15%nHA scaffolds and PCL–20%nHA scaffolds, therefore, mBMSCs had more adhesion points on them, and they had better proliferation.

### 3.7. Morphology of mBMSCs on Composite Scaffolds

**Figure 7:**
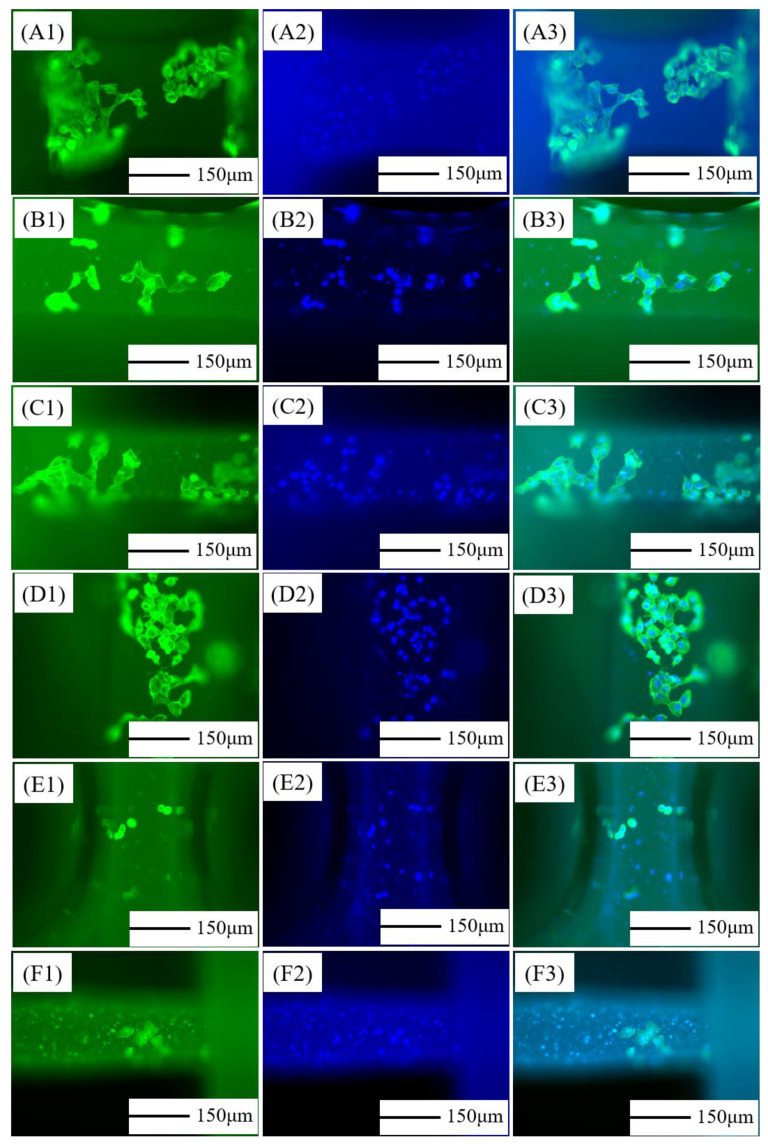
Morphology of mBMSCs cultured on PCL/nHA scaffold on the 3rd day: (**A**) Pure PCL, (**B**) PCL–5% nHA, (**C**) PCL–10% nHA, (**D**) PCL–15% nHA, (**E**) PCL–20% nHA, (**F**) PCL–25% nHA; (**A1**–**F1**) cytoplasm staining, (**A2**–**F2**) nucleus staining, (**A3**–**F3**) cytoplasm and nucleus staining.

The cytoplasm and nucleus of mBMSCs cultured for 3 days on the PCL/nHA scaffolds were stained. Figure 7(A1)–(F1) was the cytoplasm, Figure 7(A2)–(F2) was the nucleus, and Figure 7(A3)–(F3) was the superposition of the pictures of cytoplasm and nucleus, indicating the complete cell morphology. As can be seen from the figure, the cytoplasm was green, and the cell contour was clear and obvious, which was an irregular polygon. The nucleus was blue, shaped like a round ball. The cell morphology was clearly visible. The cells were attached to the surface of the scaffold, and some cells of slender pseudopodia could be seen. Pure PCL, PCL–5%nHA, PCL–10%nHA, and PCL–15%nHA scaffolds had more cells, which were scattered on the fiber surface and the morphology of the cells was full. There were few cells on the PCL–20%nHA and PCL–25%nHA scaffolds and the cells shrank into small masses. Relatively speaking, the number of cells on the PCL–15%nHA scaffold was large, and the cells were stacked together, which had better differentiation and proliferation. The PCL–15%nHA scaffold was ideal for growth support for mBMSCs.

## 4. Conclusions

In this study, PCL-based composite scaffolds with different proportions of nHA were fabricated with NFDWME technology. When the proportion of nHA was less than or equal to 25%, the composite scaffolds were easy to form. With different proportions of PCL and nHA, the physical properties of the PCL/nHA scaffolds, such as the regularity and fiber morphology were significantly different. With an increase in the nHA ratio, the regularity of the scaffolds became worse, the fiber diameter became finer, the fiber CV value became larger, and the tensile strength and fiber peeling force of the scaffolds decreased. SEM images showed that nHA dispersed on the fiber surface. The mBMSCs grew well on the Pure PCL, PCL–5%nHA, PCL–10%nHA, and PCL–15%nHA scaffolds, and proliferated on the fiber surface. The growth state of the mBMSCs on the PCL–20%nHA and the PCL–25%nHA scaffolds was poor and the number of cells was small. The presence of nHA increased the surface roughness of fibers in the composite scaffolds, which was conducive to cell adhesion and proliferation. By comparison and analysis of CCK-8 cell proliferation, live/dead cell staining and cell morphology analysis, the PCL–15%nHA scaffold had more mBMSCs on the surface and the proportion of live cells was higher than that of dead cells. The cells on the PCL–15%nHA scaffold had good activity and full growth morphology. The PCL–15%nHA scaffold was an ideal bone tissue engineering material.

## Figures and Tables

**Table 1:** Element energy spectrum of PCL/nHA scaffolds with different proportions.

Sample	C Mass/%	O Mass/%	Ca Mass/%	P Mass/%	Total
PCL–15%nHA	36.78 ± 0.64	41.04 ± 1.26	5.62 ± 0.98	16.56 ± 1.24	100
PCL–20%nHA	35.17 ± 0.59	37.70 ± 1.38	6.18 ± 0.74	20.95 ± 1.53	100

**Table 2:** Physical properties of PCL/nHA scaffolds with different proportions.

Sample	Average of Fiber Diameter/μm	CV Value of Fiber Diameter/%	Porosity/%	Tensile Strength/MPa	Fiber Peeling Force/N
Pure PCL	398	2.91	70.6 ± 0.1	2.79 ± 0.05	0.70 ± 0.02
PCL–5%nHA	292	9.34	76.1 ± 0.1	1.98 ± 0.03	0.59 ± 0.01
PCL–10%nHA	288	9.62	78.3 ± 0.1	1.92 ± 0.03	0.53 ± 0.02
PCL–15%nHA	282	9.99	78.4 ± 0.1	1.82 ± 0.02	0.47 ± 0.01
PCL–20%nHA	215	15.81	78.6 ± 0.2	1.16 ± 0.03	0.36 ± 0.02
PCL–25%nHA	212	23.77	81.3 ± 0.3	0.81 ± 0.05	0.30 ± 0.02

## Data Availability

Not applicable.
